# BMP type II receptor as a therapeutic target in pulmonary arterial hypertension

**DOI:** 10.1007/s00018-017-2510-4

**Published:** 2017-04-26

**Authors:** Mar Orriols, Maria Catalina Gomez-Puerto, Peter ten Dijke

**Affiliations:** Department of Molecular Cell Biology and Cancer Genomics Center Netherlands, Leiden University Medical Center, Leiden, The Netherlands

**Keywords:** Endothelial cell, Vascular smooth muscle cell, Signal transduction, Inflammation, Vascular remodeling and autophagy

## Abstract

Pulmonary arterial hypertension (PAH) is a chronic disease characterized by a progressive elevation in mean pulmonary arterial pressure. This occurs due to abnormal remodeling of small peripheral lung vasculature resulting in progressive occlusion of the artery lumen that eventually causes right heart failure and death. The most common cause of PAH is inactivating mutations in the gene encoding a bone morphogenetic protein type II receptor (BMPRII). Current therapeutic options for PAH are limited and focused mainly on reversal of pulmonary vasoconstriction and proliferation of vascular cells. Although these treatments can relieve disease symptoms, PAH remains a progressive lethal disease. Emerging data suggest that restoration of BMPRII signaling in PAH is a promising alternative that could prevent and reverse pulmonary vascular remodeling. Here we will focus on recent advances in rescuing BMPRII expression, function or signaling to prevent and reverse pulmonary vascular remodeling in PAH and its feasibility for clinical translation. Furthermore, we summarize the role of described miRNAs that directly target the BMPR2 gene in blood vessels. We discuss the therapeutic potential and the limitations of promising new approaches to restore BMPRII signaling in PAH patients. Different mutations in *BMPR*2 and environmental/genetic factors make PAH a heterogeneous disease and it is thus likely that the best approach will be patient-tailored therapies.

## Introduction

**Fig. 1:**
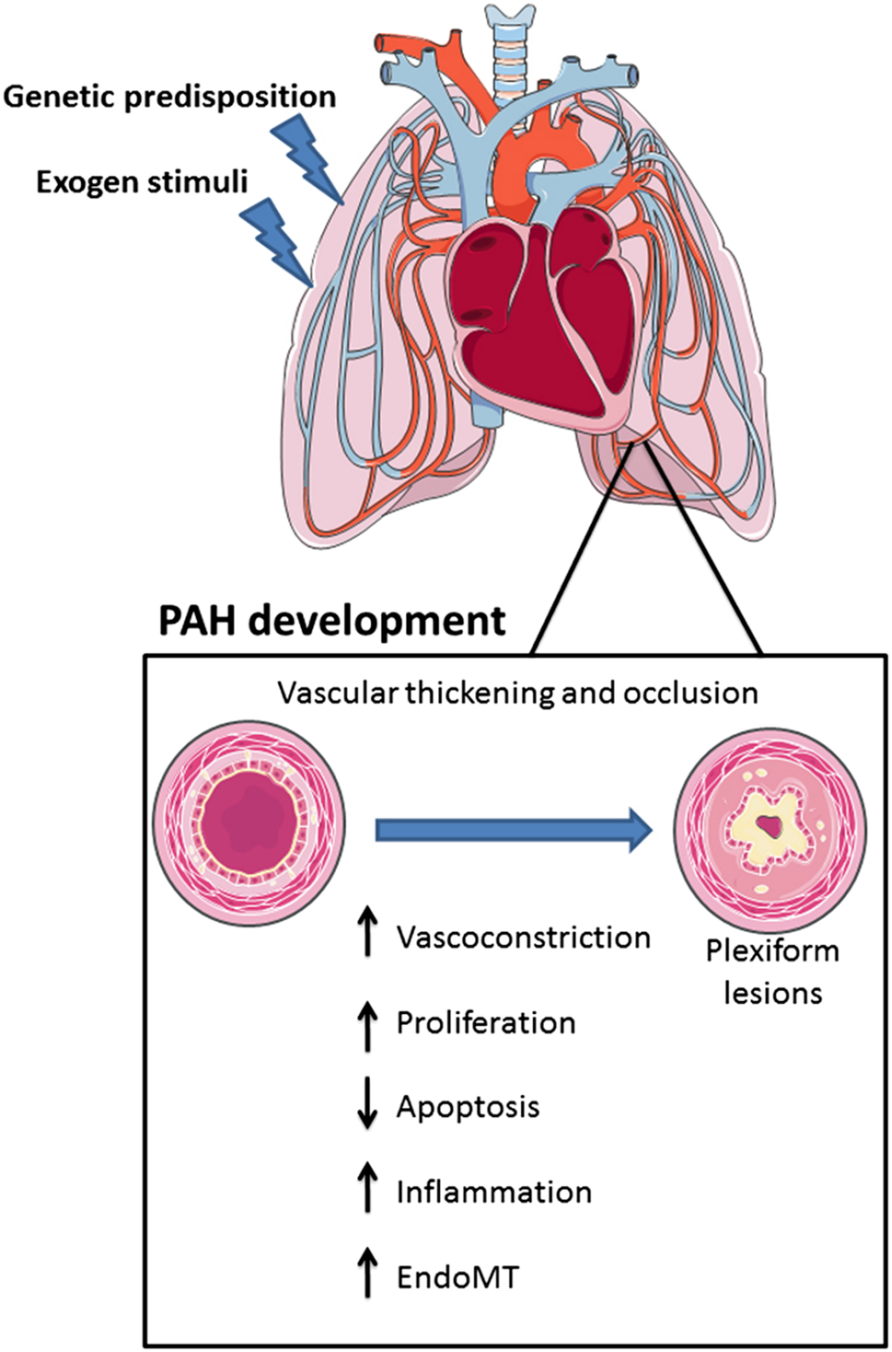
Physiopathological mechanisms of pulmonary arterial hypertension development. Presence of genetic risk factors such as *BMPR2* mutations together with exposure to deleterious environmental or biological stimuli in the lung promotes PAH. PAH development is characterized by a disturbance on the signaling pathways that control pulmonary vascular homeostasis. It results in pulmonary vascular thickening and occlusion compromising lung and heart function. *EndMT* endothelial-to-mesenchymal transition

Pulmonary arterial hypertension (PAH) is a chronic disease characterized by a progressive elevation in mean pulmonary arterial pressure (mPAP >25 mmHg) leading to right heart failure and death [1]. PAH is characterized by abnormal remodeling of small peripheral lung vasculature resulting in progressive occlusion of the artery lumen. In addition, at late stages, so-called plexiform lesions are found, which are complex vascular formations originating from abnormal endothelial cell (EC) proliferation and vascular smooth muscle cell (SMC) hypertrophy [2]. The basic pathogenic mechanisms underlying this disease include vasoconstriction, intimal proliferation, and medial hypertrophy. These processes are accompanied by illicit recruitment of inflammatory cells which release factors enhancing cell proliferation and elastin fibers degradation [3, 4] (Fig. 1).

More than 70% of patients with familial PAH and 20% of idiopathic PAH show heterozygous mutations in the bone morphogenetic protein type II receptor (BMPRII) [5–8]. BMPRII is a transmembrane serine/threonine kinase receptor of the bone morphogenetic protein (BMP) pathway which is essential for embryogenesis, development, and adult tissue homeostasis. Upon BMP-induced heteromeric complex formation of BMPRII with BMP type I receptor (BMPRI), BMPRII activates BMPRI by phosphorylation. Thereafter, the activated BMPRI propagates the signal into the cell through phosphorylation of the SMAD1/5/8 transcription factors.

In PAH, over 300 mutations have been found in the *BMPR2* gene. These mutations target sequences that encode the ligand binding and kinase domain and the long cytoplasmic tail; the mutations compromise BMPRII function [9]. Although the BMPRII pathway is essential for vascular homeostasis and there is a strong correlation between *BMPR2* mutations and PAH, the incomplete penetrance of BMPRII mutations (20–30%) suggests that other genetic and environmental factors contribute to the disease. Among them, *BMPR2* alternative splicing plays a role in PAH penetrance. One *BMPR2* splice variant lacks exon 12, which is the largest exon of the gene and encodes the cytoplasmic tail. It has been shown that carriers of this variant are more prone to develop PAH through a dominant-negative effect (DN) effect on wild-type BMPRII [10]. Furthermore, there are mutations in other genes in the BMP pathway, which further strengthens the notion of a causal role for this pathway in PAH [11]. Moreover, the co-existence of modifier genes, infections, toxic exposure, inflammation, or alterations in estrogen metabolism has been described [11–14] and some of them were found to downregulate BMPRII expression. For example, pro-inflammatory cytokines such as tumor necrosis factor α (TNFα) and Interleukin 6 induce the expression of miRNAs that inhibit BMPRII expression [15]. Furthermore, BMPRII is essential for maintaining the barrier function of the pulmonary artery endothelial cell lining and BMPRII deficiency increases endothelial inflammatory responses thereby contributing to adverse vascular remodeling [16–18].

Current therapeutic options for PAH are limited and focused mainly on reversal of pulmonary vasoconstriction and proliferation of vascular cells through targeting of prostacyclin (PGI_2_), endothelin, or nitric oxide pathways [19]. Although these treatments can relieve disease symptoms and slow down its progression, PAH remains a progressive lethal disease. Abundant research over the past decade has improved our understanding of the molecular mechanisms underlying PAH progression revealing novel potential therapeutic interventions [20–22]. Among them there are several anti-proliferative strategies including cell cycle inhibitors (e.g., mTOR inhibitor rapamycin) and anti-apoptotic drugs (e.g., surviving inhibitors) [23]. Furthermore, based on the fact that Rho and ROCK mediate smooth muscle cell proliferation in a serotonin-BMPR-dependent pathway, Rho-kinase inhibitors have been also considered [23, 24]. Although several drugs with possible benefit in PAH have been identified, only very few have been approved for use in the clinic due to toxicity or lack of clinical efficacy. This review will focus on recent advances on the rescue of BMPRII expression, function, or signaling to prevent and reverse pulmonary vascular remodeling in PAH. We will discuss data on the in vitro efficacy of the different approaches together with the physiological outcomes in pre-clinical models and their feasibility for clinical translation.

## BMP signaling in vascular biology and PAH

**Fig. 2:**
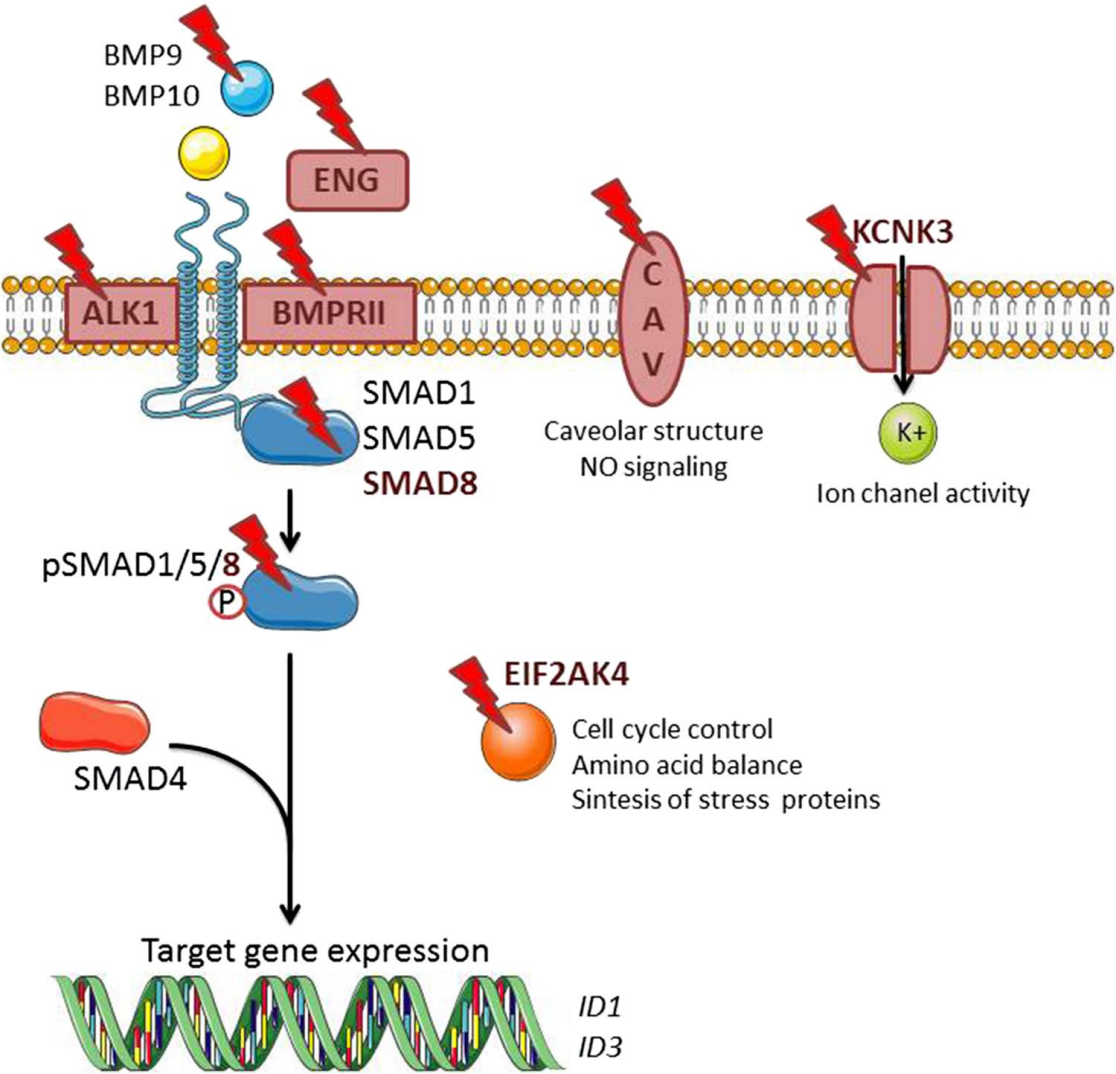
BMP signaling in endothelial cells. BMP9 and BMP10 present in the circulation initiate signaling by binding and bringing together BMPRII and ALK1. BMPRII phosphorylates ALK1 which then propagate the signal through phosphorylation of SMAD1/5/8. Subsequently, SMAD4 forms a complex with SMAD1/5/8, which translocates to the nucleus regulating the expression of target genes such as ID1 and ID3. Known gene mutations associated with PAH are highlighted in red. It includes mutations in BMP signaling components (*GDF2, BMPR2, ALK1, SMAD8*, and *ENDOGLIN*) as well as recently discovered non-directly related BMP genes (*CAV1, KCNK3,* and *EIF2AK4*). *CAV* caveolin, *EFI2AK4* eukaryotic translation initiation factor 2α kinase 4, *ENG* ENDOGLIN, *ID* inhibitor of DNA binding, *KCNK3* potassium channel subfamily K member 3

BMPs belong to the multifunctional transforming growth factor-β (TGF-β) family of secreted dimeric cytokines. The effects of BMPs are highly dependent on cellular context [25]. In general, BMPs control cellular proliferation, differentiation, and apoptosis, and play an important role in embryonic development and maintaining tissue homeostasis [26]. Therefore, perturbation of BMP signaling may lead to skeletal diseases, vascular diseases, and cancer [27]. BMPs can be subdivided into four subgroups based on their sequence similarity and cell surface receptor affinities: BMP2/4, BMP5/6/7/8, BMP9/10, and growth and differentiation factor (GDF)-5/6/7 [28, 29]. BMPs signal via hetero-tetrameric combinations of type I receptors (activin receptor-like kinase (ALK)1, ALK2, ALK3, or ALK6) and BMP type II receptors (BMPRII) and activin type II receptor (ACVRII)A or ACVRIIB complexes [30, 31]. Both, type I and type II receptors have a similar structure encompassing a short extracellular domain, a single transmembrane domain and an intracellular domain with intrinsic serine-threonine kinase activity. In the vascular endothelium, BMP signaling is mainly activated by BMP2, 4, 6, 9, and 10 [32]. BMP2 and BMP4 bind preferentially to the BMPRII in complex with ALK3 or ALK6. BMP6 binds to the ACVRIIA-ALK2 complex, while BMP9 and BMP10 bind to BMPRII or ACVRII in combination with ALK1 or ALK2. Whereas BMPRII is a specific receptor for BMPs, ACVRIIA and ACVRIIB can also interact functionally with other ligands, such as activins, myostatin, and nodal. Interestingly, ALK2 and 6 are widely expressed in various cell types, while ALK1 has a more selective expression pattern and is mainly restricted to ECs. After BMP binding and receptor complex formation, the type II receptor kinase phosphorylates the type I receptor on serine and threonine residues in the glycine-serine rich (GS)-domain causing its activation and subsequent phosphorylation of the receptor-associated R-SMAD1, 5, and 8 effector proteins. The R-SMADs that are activated by TGF-β type I and activin type I receptor (i.e., ALK5 and ALK4, respectively) are SMAD2 and SMAD3 and these are distinct from BMP R-SMADs. Activated R-SMAD 1, 5, or 8 forms a hetero-oligomeric complex with common mediator co-SMAD4. This complex translocates to the nucleus and regulates the expression of target genes by binding to specific enhancers/promoters upstream of these target genes [30, 33, 34] (Fig. 2). Besides canonical BMP receptor/SMAD signaling, activated BMP receptors can initiate non-SMAD signaling pathways such as ERK, JNK, p38 MAP kinases, and the phosphatidyl inositol 3 kinase (PI3K)/AKT pathways [35–37]. These non-SMAD pathways are also important for diversifying and modulating the canonical SMAD signaling pathways that are activated by the BMP receptors [38, 39]. In addition, BMP activity is also regulated by several extracellular modulators, including BMP binding proteins NOGGIN, CHORDIN, and FIBULINs. Co-receptors such as ENDOGLIN, BETAGLYCAN, and DRAGON family members may also modulate the interactions between BMPs and BMP receptors [40, 41]. Moreover, intracellular kinases/phosphatases and other binding proteins have been identified as regulators of the trafficking, subcellular localization, stability, and function of BMP receptors and SMADs [26].

Genetic depletion of different components of the BMP signaling cascade leads to embryonic death due to cardiovascular malformations and abnormal vascular remodeling. BMP signaling plays an important role in vasculogenesis (de novo formation of blood vessels from undifferentiated mesodermal cells) and angiogenesis (formation of new blood vessels from the existing vasculature). In this light, it is not surprising to discover that, besides PAH, dysfunction of BMP signaling has been found to be associated with other vascular diseases including hereditary hemorrhagic telangiectasia, cerebral cavernous malformation, atherosclerosis, and vascular calcification among others [42]. Furthermore, BMPRII downregulation has been found to be involved in pancreatic and lung fibrosis [43, 44].

Blood vessels are composed of three layers: the tunica adventitia consisting of fibroblasts and associated collagen fibers; the tunica media composed of SMCs; and the tunica intima consisting of ECs coating the interior surface [45, 46]. ECs, SMCs, and fibroblasts have been found to play a role in the pathogenesis of PAH. Abnormal EC proliferation resulting in the formation of plexiform lesions has been frequently described in many PAH cases [47]. In addition, pulmonary arterial SMCs show an increased proliferation and decreased apoptosis leading to vessel wall thickening and vascular remodeling (Fig. 1). The close interaction between ECs and SMCs has been found to be involved in vessel formation and maintenance. For instance, endothelial derived factors, like endothelin and angiotensin II, affect SMCs which increases vascular tone. Similarly, nitric oxide and PGI_2_ secreted by ECs modulate the vasodilator response of SMCs [48]. In particular, PGI_2_ has been found to be reduced in PAH patients [49].

BMP signaling is known to control cell migration, proliferation, and apoptosis in ECs and SMCs [45]. BMP9 and BMP10 are present in the circulation and play an important role in the vasculature. Their associated receptors BMPRII and ALK1 and co-receptor ENDOGLIN are predominantly expressed on ECs [50, 51] and together can modulate the ability of ECs to migrate and proliferate [27]. Furthermore, BMPRII is also expressed in vascular SMCs where it has been shown to be necessary for the control of proliferation and differentiation [52]. Besides mutations in the *BMPR2* gene, mutations in the genes of other BMP signaling components (such as *GDF-2*, *ACVRL1, ENDOGLIN*, and *SMAD8*) have also been linked to PAH development [11, 53–59]. This association reinforces the importance of BMP signaling in the control of vascular homeostasis, and suggests that there is a causal link between perturbation of canonical BMP/SMAD signaling and PAH. In support of this view, recent, new DNA sequencing techniques helped to identify new gene mutations associated to PAH [*Caveolin-1* (*CAV1*), *KCNK3*, and *EIF2AK4*] [60, 61] (Fig. 2).

The abnormal vascular remodeling that characterizes PAH involves an accumulation of α-smooth muscle, actin-expressing mesenchymal-like cells indicating that the endothelial-to-mesenchymal transition (EndMT) may be involved in the pathogenesis of the disease [62]. In addition, BMPRII reduction in pulmonary artery endothelial cells (PAECs) has been found to promote the trans-differentiation of epithelial cells into motile mesenchymal cells via the transcription factors high-mobility group protein (HMGA)1 and its target SLUG [63].

## Animal models of PAH

PAH has a complex etiology and pathobiology with many factors contributing to its development [64]. A variety of pre-clinical rodent models have been used to study the underlying pathophysiological mechanisms and to test novel therapeutic strategies for PAH. A proper model should be reproducible, inexpensive, and faithfully reproduce (in a defined period) the basic features of PAH such as complex destructive neointimal lesions and right ventricle (RV) dysfunction and failure. To date, there is no model that recapitulates all of the pathophysiological mechanisms and the clinical course of human PAH. For instance, in the chronic hypoxic exposure or monocrotaline (MCT)-induced rat models, pulmonary hypertension rarely develops with the same severity observed in humans perhaps due to the absence of obstructive intimal lesions in the peripheral pulmonary arteries [65, 66]. Furthermore, the chronic hypoxia model does not lead to RV failure, while MCT injection causes myocarditis affecting both ventricles and causing liver and kidney damage [67]. These limitations may explain why it is difficult to translate the reversal of PAH in animal models by several experimental compounds into therapies for PAH patients.

In recent years, second-generation animal models have been established based on the combination of multiple triggers: MCT plus pneumonectomy, MCT plus chronic hypoxia, and SU5416 plus chronic hypoxia. To circumvent the problem of the embryonic lethality of *BMPR2* knock-out mice, switchable rodent models have been developed, by means of *BMPR2* conditional knock-out, whereby the mutation can be activated after birth [68–70]. Moreover, genetic rodent models have been developed including overexpression of interleukin-6. These new models closely mimic the features and the severity of human PAH although not completely [71]).

## Restoring BMPRII signaling as a therapeutic strategy

While hereditary PAH have been linked to heterozygous mutations in the *BMPR2* gene, non-genetic forms of PAH show a reduction in BMPRII levels and activity [9]. Consistent with this, heterozygous *BMPR2* deletion in PAECs and pulmonary artery smooth muscle cells (PASMCs) mimics the PAH phenotype [69, 72]. Furthermore, mice expressing a dominant-negative BMPRII (lacking an intracellular domain) in vascular SMC, develop vascular lesions in the lungs [68, 72].

**Fig. 3:**
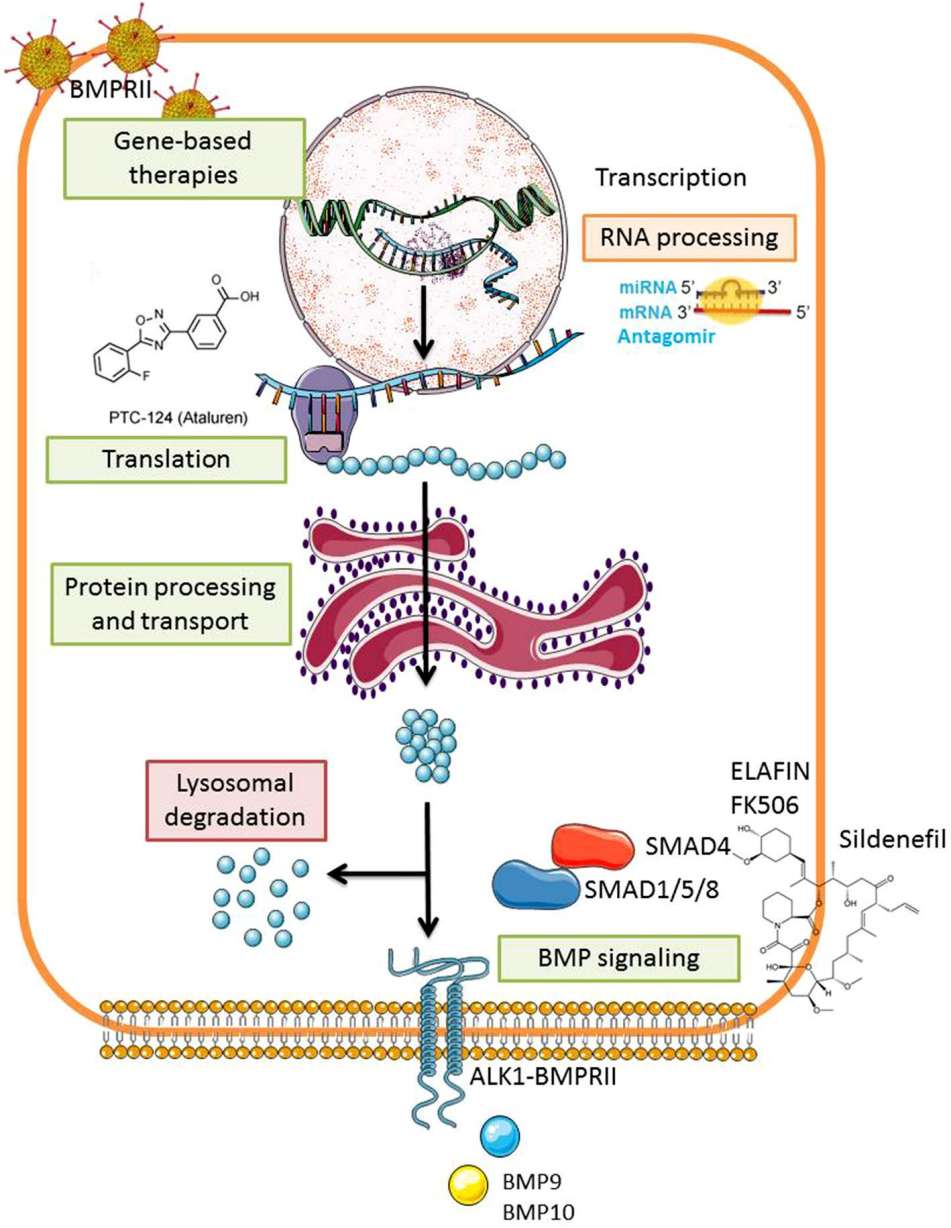
Rescuing the BMPRII signaling pathway in pulmonary arterial hypertension. Modulation of BMPRII signaling is considered a promising therapeutic approach for PAH. This could be achieved by different methods aiming to increase the amounts of BMPRII present in the cell or to trigger BMP signaling. These approaches include exogenous BMPRII delivery, inhibition of miRNAs negatively regulating BMPRII stability and translation, inhibition of lysosomal degradation, and delivery of exogenous BMP ligands or BMP coactivators among others

There is strong evidence suggesting that BMPRII signaling has a protective role in the vascular wall by promoting the survival of PAECs, inhibiting PASMCs proliferation and triggering anti-inflammatory responses [17, 73, 74]. Based on this, modulation of BMPRII signaling is considered a promising therapeutic approach for PAH. Importantly, the rescue of BMPRII expression may not exclusively benefit PAH patients but also patients suffering from pancreatic and lung fibrosis where BMPRII deficiency has been implicated [43, 44]. BMPRII restoration can be targeted at different levels: genetic-based therapies, transcriptional and translational regulation, protein activity, and processing as well as SMAD downstream signaling modulation [27, 75, 76] (Fig. 3).

### Genetic-based therapies

#### Exogenous *BMPR2* gene delivery

One strategy to treat PAH patients is to rescue BMPRII expression through gene therapy targeting ECs. In pre-clinical models, this was explored by Reynolds et al., who administrated a vector inducing BMPRII expression via tail-vein injection. The *BMPR2* encoding virus targets the pulmonary endothelium by binding to the highly expressed pulmonary endothelial angiotensin-converting enzyme (ACE) using a bi-specific conjugate antibody. This *BMPR2* adenoviral vector restored BMPRII protein levels in human microvascular PAECs and attenuated the PAH phenotype in a chronic hypoxia model and MCT-treated rats [77, 78]. Furthermore, BMPRII overexpression in lung tissue was shown to reverse the imbalance between BMPRII and TGFβ signaling thus restoring normal levels of pSMAD 1/5/8 and the activation of PI3K and p38 MAP kinase [79]. In contrast, BMPRII administration via an aerosol route targeting PASMCs did not improve the PAH phenotype when tested in the MCT model [80]. The later result highlights the importance of BMPRII signaling in ECs but not SMCs. However, further investigations will be required to elucidate precisely how spatio-temporal control BMPRII overexpression might provide therapeutic benefit in the context of *BMPR2* mutations. It should be noted that adenoviral vectors are only capable of transient gene expression since the delivered gene is not integrated into the host chromosome. Stable integration can be achieved and lentiviral vectors are potentially an attractive vehicle to deliver longer term transgene expression since they integrate into the genome and they can infect non-proliferating cells, when compared to retroviral vectors. An important potential limitation of this approach is that integrating vectors may generate gene mutations upon insertion and newer advances regarding self-inactivating vectors are needed [81, 82]. Adeno-associated virus and helper dependent adenoviral vectors (the latest generation of recombinant adenoviral vectors) are a promising alternative since they deliver longer durations of transgene expression when compared to the first-generation vectors. Moreover, they show neither long-term adverse effects in liver nor an immunological response [83, 84]. Taken together, exogenous *BMPR2* delivery is a possible therapy for PAH, but further improvements in vector technology are required to translate this approach to the clinic for the treatment of pulmonary vascular disease.

### Transcriptional regulation

#### miRNA targeting BMPRII

**Fig. 4:**
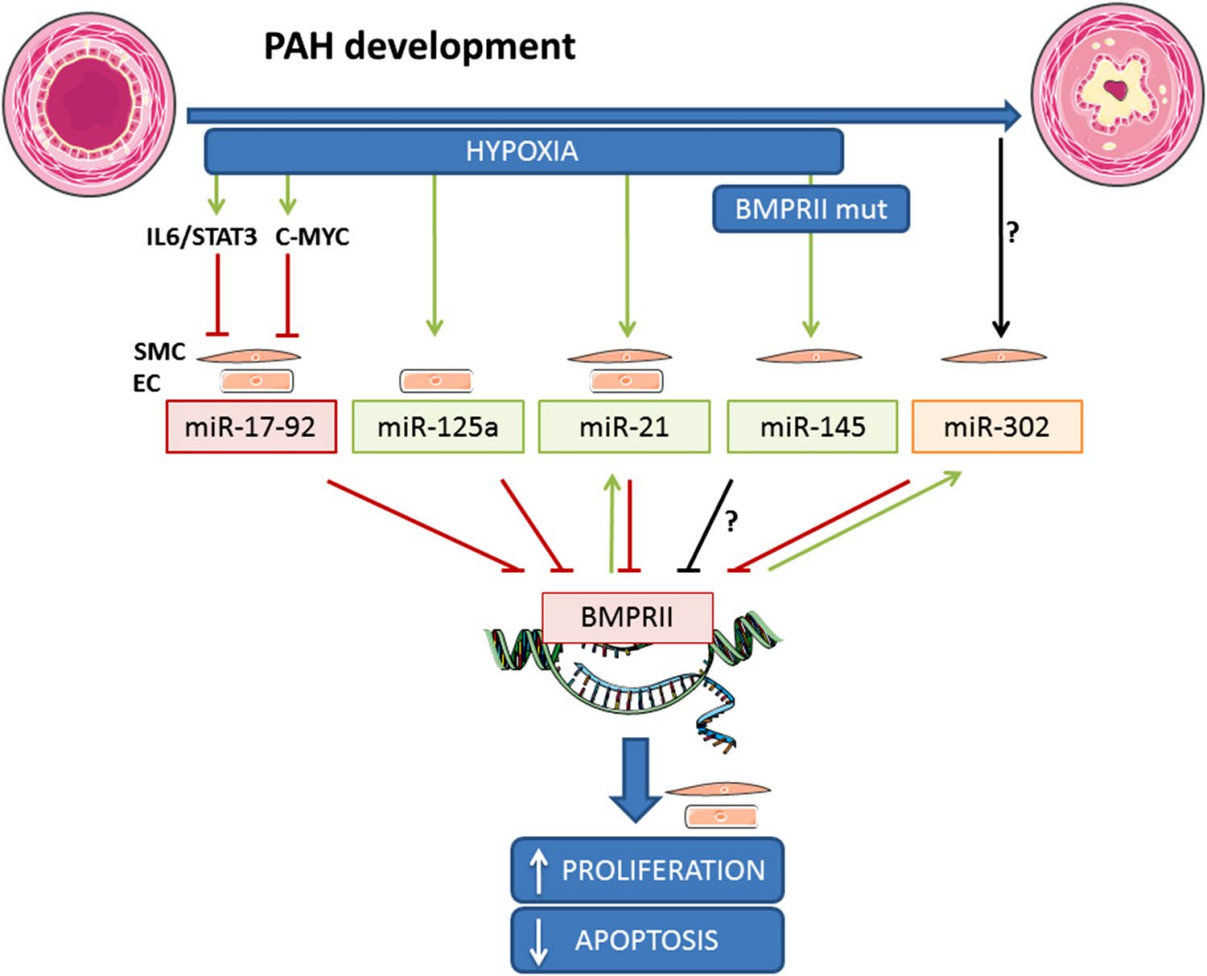
miRNAs targeting BMPRII in the vascular wall. The illustration shows hypoxia and BMPRII mutations as regulators of miRNAs expression in endothelial or smooth muscle cells. These miRNAs negatively regulate BMPRII expression resulting in increased cell proliferation and impaired apoptosis. *Green arrows* indicate activation, *red arrows* represent inhibition, and black arrows correspond to unknown regulation. *EC* endothelial cells, *IL* interleukin, *miR* micro RNA, *mut* mutant, *SMC* smooth muscle cell, *STAT* signal transducer, and activator of transcription

In recent years, there has been an increasing interest in the role of epigenetics in the development of PAH [85, 86]. Epigenetics refers to heritable changes in gene expression that do not involve alterations in the DNA sequence. miRNAs are small non-coding RNAs that negatively, post-transcriptionally regulate the expression of target genes by interfering with both the stability of the target transcript as well as its translation. miRNAs have emerged as essential players in the development (and diseases) of the cardiovascular system. They also play an important role in vascular remodeling [87, 88]. miRNAs are expressed in the vasculature and are essential for the regulation of vessel function. Many miRNAs control proliferation, differentiation, and apoptosis of ECs and SMCs by targeting components of the TGF-β/BMP signaling pathways. Several miRNAs, such as miR-145, miR-21 and the miR17/92 cluster, have been associated with the disrupted BMPRII pathway in PAH and can explain the incomplete penetrance of *BMPR2* mutations [89–91]). Figure 4 and Table 1 provides an overview of currently described miRNAs that target *BMPR2* expression in vascular cells. In addition, Table 1 shows a list of other miRNAs that are predicted to target *BMPR2* in silico.

**Table 1:** miRNA targeting BMPRII expression

MicroRNA	Cell type	Function	Model	Expression	References
miR-17/92	EC	Interleukin-6 modulates the expression of the BMPRII through a novel STAT3–microRNA Cluster 17/92 pathway	PAEC	∃	Brock et al. [13]
	SMC	Inhibition of miR-17 enhances BMPRII expression and improves heart and lung function in experimental PH	PASMC Hypoxia-induced PH mice MCT-induced PH rats	?	Pullamsetti et al. [164]
miR-20A	SMC	Treatment with antagomiR-20a restores functional levels of BMPRII in pulmonary arteries and prevents the development of vascular remodeling	PASMC Hypoxia-induced PH mice	?	Brock et al. [165]
miR-21	EC	Hypoxia and BMPRII signaling independently upregulate miR-21. In a reciprocal feedback loop, miR-21 downregulates BMP receptor type II expression	PAEC Several rodent models of PH miR-21-null mice	#	Parikh et al. [166]
	SMC	BMPRII was downregulated in PASMCs overexpressing miR-21	PASMC Hypoxia-induced PH mice	#	Yang et al. [167]
miR-125	EC	Inhibition of miR-125a resulted in upregulated BMPRII expression accompanied by increased proliferation of EC	PAEC Hypoxia-induced PH mice Plasma PAH patients	#	Huber et al. [168]
miR-143/145	SMC	miR-145 expression is increased in primary PASMCs cultured from patients with BMPRII mutations and in the lungs of BMPRII-deficient mice	PASMC Hypoxia-induced PH mice BMPRII R899X knock-In Mice miR-145 knock-Out Mice Lugn tissue PAH patients	#	Caruso et al. 2012 [169]
miR-302	SMC	Inhibition of miR-302 by BMP4 increases BMPRII expression and facilitates the BMP signaling pathway	PASMC	?	Kang et al. 2012 [170]
miR-181c	cardiac	Increased miR-181c expression in human cardiac samples from individuals with ventricular septal defects (VSD) was correlated with downregulated BMPRII levels	Human VSD cardiac samples	#	Li et al. [171]

Currently, there are different technologies to inhibit aberrantly overexpressed miRNAs including the use of antisense oligonucleotides, masking, sponges, erasers, or decoys [92, 93]. In addition, administration of miRNA mimics can enhance the expression of downregulated miRNAs [94]. These strategies are still under development and more research is needed to establish how modulation of miRNA function could offer therapeutic benefits in a clinical setting while avoiding off-target effects, especially in the liver, where systemically administrated miRNAs or modulating compounds preferentially accumulate [95].

The delivery routes mostly used to target lung disease are local intranasal and intra-tracheal administration. These naked miRNAs are directly delivered into the lung with minimal systemic side effects [96]. Nevertheless, this method remains ineffective and challenging due to the complexity of the lung [97]. Recent advances in delivery strategies, such as the use of liposomes, nanoparticles, or virus, combined with improvements in chemically modifying miRNAs, represent promising strategies to improve lung miRNA delivery [98]. More than 20 miRNAs are currently in clinical trials, several in Phase III stage, highlighting the potential of miRNA therapeutics to restore BMPRII in PAH [99]. To date, the potential for miRNAs as a therapeutic tool is relatively limited. Further studies focusing on the specificity, safety, efficiency, and stable systemic delivery of miRNAs into target cells or tissues will improve the process of translating these findings to the clinic.

### Translational regulation

#### Read-through premature STOP codons

Most *BMPR2* mutations (~70%) are non-sense mutations (frame-shift deletions and insertions) generated by the insertion of a premature termination codon (PTC) resulting in truncated reading frames which produce non-functional proteins [7]. To prevent formation of truncated proteins, mutated transcripts are directly degraded through non-sense mediated decay (NMD) resulting in insufficient amounts of the functional protein, which is produced only by the wild-type allele (haplo-insufficiency) [100, 101]. NMD usually does not completely reduce the levels of mutated transcripts and as a result truncated proteins persist, and may exert a DN effect [102].

An approach aimed to correct these types of mutations consists on the induction of PTC read-through. Read-through of truncated mutations by aminoglycoside antibiotics, such as Gentamicin, has been extensively studied and has reached the clinical trial stage for genetic disorders such as cystic fibrosis [103] and Duchenne muscular dystrophy [104–110]. Aminoglycoside antibiotics bind to the decoding site of ribosomal RNA and eliminates the PTC by incorporating an amino acid to generate full-length proteins [111]. Importantly, this read-through function of aminoglycosides does not affect normal translation because of the presence of upstream and downstream regulatory sequences around a normal termination codon that ensure optimal efficiency of termination [112]. Gentamicin treatment has been tested in lymphocytes derived from two PAH patients with PTC mutations [113, 114]. The results demonstrated increased amounts of full-length BMPRII protein, a reduction in the mutated BMPRII product and enhanced BMPRII downstream signaling. Although aminoglycosides are commonly used in the clinic to treat infections and are safe when administered directly to the lung by inhalation, several side effects of long-term treatments and/or high concentrations of the drug have been shown.

Recently, a high-throughput screening for compounds that suppress non-sense mutations identified a new small molecule named Ataluren (PTC124) which mediates read-through of premature stop codons without acute side effects [115]. Aldred et al. have demonstrated that after Ataluren treatment, BMPRII protein levels were normalized and BMP-dependent phosphorylation of the downstream target R-SMADs was increased in PAECs and PASMCs from PAH patients. In addition, the hyper-proliferative phenotype of these cells was reversed even in the presence of significant non-sense mediated mRNA decay. Although, further studies, including animal models, are required to explore the relevance of Ataluren in vivo in a PAH context, the compound has emerged as a promising therapeutic strategy for a subset of PAH patients.

### Protein processing regulation

#### Rescue of BMPRII trafficking

30% of *BMPR2* mutations are missense mutations leading to single amino acid substitutions in a conserved domain affecting the overall function of the protein [7]. Mutations resulting in the substitution of cysteine residues in the ligand binding and kinase domains disrupt protein folding and trafficking of BMPRII to the cell surface leading to retention of the mutant receptor in the endoplasmic reticulum (ER) [113, 116]. A potentially promising therapeutic strategy to increase the expression of BMPRII at the plasma membrane is to enhance the activity of chaperones which facilitate protein folding and trafficking. This can be done by means of chemical chaperones such as sodium phenyl-butyrate (4-PBA), probenecid, and tauroursodeoxycholic acid (TUDCA). These have been shown to improve protein trafficking via several distinct mechanisms [117–126]. Different groups have demonstrated that treatment with chemical chaperones can partially restore cell surface expression of BMPRII in ECs. As a result, BMP-induced SMAD 1/5/8 phosphorylation and the expression of the target gene ID1 is restored [127–129]. These agents are showing promise in clinical trials for other diseases caused by misfolded proteins, such as cystic fibrosis. Since the currently used chemical chaperones are federal drug administration (FDA) approved drugs, there is an immediate translational potential to treat PAH patients [118, 130–133]. However, further in vivo studies are required to test the viability of this approach.

Even though chemical chaperones have the potential to rescue the BMPRII mutants which are retained in the ER, it remains to be investigated whether the amount of BMPRII reaching the plasma membrane is enough to induce a clinically relevant effect. Also, BMPRII with a protein-folding defect expressed at the cell surface may have a dominant-negative activity and adverse effects on BMP signaling [127]. Moreover, patients harboring missense mutations that affect the activity of the receptor (kinase domain) may not benefit from this therapeutic strategy. Further research, taking this mutation variability into account, is required to determine which patients might benefit from this approach.

#### Inhibition of lysosomal degradation

The deciphering of mechanisms which regulate cell surface expression levels of BMPRII are of potential clinical importance, particularly those mechanisms that prevent its rapid turnover and thereby restore downstream BMPRII signaling and function. In this context, several studies focused on the potential of targeting the degradation of BMPRII by preventing lysosomal degradation [134]. Durrington et al. have demonstrated that after Kaposi sarcoma-associated herpesvirus infection, BMPRII is ubiquitinated by K5 (membrane-associated RING E3 viral ubiquitin protein ligase) leading to lysosomal degradation in primary cultured pulmonary vascular cells [134]. In addition, cells treated with the lysosomal inhibitor concanamycin A exhibit increase levels of BMPRII. Furthermore, through siRNA screening of the NEDD4-like family E3 ubiquitin protein ligases, it was found that knockdown of *ITCH* expression resulted in increased BMPRII protein levels [134]. Whether ITCH ubiquitinates BMPRII, leading to lysosomal degradation, has yet to be investigated. Satow et al. have demonstrated that BMPRII is degraded via the proteosomal pathway in HEK 293T cells, when it is associated with Dullard phosphatase [135]. This might suggest that more than one mechanism accounts for BMPRII proteasome-mediated degradation. It is noteworthy that Satow et al. used a BMPRII overexpression system, whereas Durrington et al. studied the degradation of endogenous BMPRII [134]. Different membrane trafficking pathways such as endocytosis, phagocytosis, micropinocytosis, and autophagy, use lysosomes for the digestion of diverse macromolecules [136]. Caveolae-mediated endocytosis affects multiple cellular signaling pathways by the redistribution of transmembrane receptors and receptor-ligand complexes [137–139]. BMPRII localization has been found to be regulated by CAV1 in vascular SMC [137]. Recently, ELAFIN (endogenous serine protease inhibitor) treatment has been shown to prevent and reverse PAH in the SU-hypoxia rat model. This occurs via elastase inhibition and by promoting the interaction of BMPRII with CAV1. Interestingly, when ELAFIN was combined with BMP9, there was enhanced co-localization of CAV1 and BMPRII on PAEC surfaces, which led at an increase in BMP9-dependent SMAD1/5 phosphorylation and induction of ID1 [137]. Furthermore, transgenic mice overexpressing human ELAVIN in the cardiovascular system (by placing ELAVIN expression under the control of the pre-proendothelin-1 promoter), exhibited reduced SMC proliferation and medial/intimal thickening after carotid artery wire injury [140] and were protected from hypoxic pulmonary hypertension [141]. In agreement with this, peptidyl trifluoromethylketone serine elastase inhibitors such as M249314 or ZD0892, have been used to prevent and reverse PAH in the MCT rat model [142]. However, the clinical use of these compounds was not pursued due to hepatotoxicity. ELAFIN has been shown to inhibit myocardial ischaemia-reperfusion injury induced during coronary artery bypass graft surgery [143]. Even though ELAFIN infusion was safe and resulted in >50% inhibition of elastase activity in the first 24 h, myocardial injury was not reduced after 48 h. Based on the biology of ischemia–reperfusion injury and PAH, we believe it is worth testing whether ELAFIN together with BMP9 could reverses PAH in patients.

Interestingly, autophagy has also been found to be involved in PAH. Autophagy (literally, “self-eating” in Greek) is a highly regulated catabolic process that involves sequestration and lysosomal degradation of cytosolic components such as dysfunctional organelles, misfolded proteins, lipid droplets, and invading pathogens [144]. Autophagy can be considered to be a general housekeeping mechanism maintaining the integrity of intracellular organelles and proteins. It is also triggered during development, differentiation, infection, and stress conditions. Thus, autophagy can be activated in the presence of damaged organelles, protein aggregates, intracellular pathogens, hypoxia, amino acid starvation, reactive oxygen species, and DNA damage [145]. Long et al. have shown that in rats suffering from PAH induced by MCT treatment, there is increased autophagy together with a decrease of BMPRII protein expression [146]. Moreover, inhibition of autophagic degradation by the lysosomal inhibitors chloroquine and hydroxychloroquine [147] prevents the development of PAH as well as its progression. The authors demonstrated that chloroquine and ATG5 (an autophagy protein involved in the elongation and closure of the autophagosomal membrane) knockdown inhibited proliferation and increased apoptosis of PASMCs and these effects correlated with increased levels of BMPRII via lysosomal inhibition. Although autophagy seems to be involved in the degradation of BMPRII, the exact mechanism by which this takes place has yet to be elucidated. Chloroquine and hydroxychloroquine have been widely utilized in malaria prophylaxis [148]. They have also been used to treat rheumatoid arthritis and lupus erythematosus (as anti-inflammatory agents) [148]. Since inflammation is thought to be a crucial second hit in PAH [149], these drugs might be effective at inhibiting PAH progression by impairing the degradation of BMPRII as well as inhibiting the inflammatory response. However, it is important to keep in mind that since lysosomal degradation is a ubiquitous cellular mechanism for regulating protein processing, this approach can lead to widespread and non-specific off-target effects independent of BMPRII signaling. Therefore, an improved understanding of the molecular mechanisms underlying BMPRII turnover is required for the development of more directed interventions.

### BMPRII signaling regulation

#### Delivery of exogenous BMP ligand

As mentioned previously, BMP signaling in the vascular endothelium is mainly activated by BMP2, 4, 6, 9, and 10 [32]. In particular, BMP9 and BMP10 appear to play an important role in the vasculature due to their presence in the circulation and based on the fact that they are known to signal through receptors expressed on the endothelium, such as ALK1 and BMPRII or ACVRIIB. Therefore, the stimulation of BMP signaling with exogenous recombinant ligand is an interesting approach for PAH treatment [11, 150]. Long et al. have shown that BMP9 prevents apoptosis and enhances the integrity of ECs in PAECs and blood outgrowth ECs from PAH patients. Furthermore, therapeutic BMP9 delivery prevents and reverses PAH in several mouse models [70]. BMP10 is the least studied BMP ligand; however, it may present a better treatment than BMP9 since it binds to ALK1 and BMPRII with higher affinity and because of its lack of osteogenic activity in vitro [151]. Further studies have to be performed to evaluate the delivery strategies, efficiency, and potential side effects of BMP9 and BMP10 in vivo. Finally, the development of a small peptide mimetics of BMP9 or BMP10, with an increased affinity for the receptor, is a theoretical alternative for efficiently activating BMP signaling and thereby reversing PAH [150].

#### Enhance downstream SMAD signaling

An additional approach to reverse the effect of mutant BMPRII is use small molecules to enhance signaling of the wild-type functional proteins. Sildenafil is a phosphodiesterase type-5 (PDE5) inhibitor currently used in the clinic for PAH treatment [152–154]. Its mode of action is to block the degradation of cyclic guanosine monophosphate (cGMP) resulting in corrective vasodilatory and anti-proliferative effects in the arterial wall [155]. Furthermore, it has been described that protein kinase G (PKG) activated by cGMP, is a modulator of BMP signaling [156] and that PASMCs expressing a BMPRII mutant, showed an increase in BMP signaling after Sildenadil treatment via a cGMP/PKG-dependent mechanism. In addition, in vivo studies confirmed that Sildenafil treatment enhanced BMP signaling and partially reversed PAH development in the MCT rat model [157, 158]. Although Sildenafil therapy during 12 weeks improves multiple clinical symptoms in PAH patients, it appears to have no effect on reducing either mortality or serious adverse events [159]. Furthermore, the long-term efficiency and safety of Sildenafil therapy in PAH requires further studies based on large and well-designed clinical trials [159].

Another promising strategy is to identify compounds in drug libraries that activate BMP/SMAD signaling. FK506 (Tacrolimus) was identified as the best BMP coactivator among 3756 FDA-approved drugs and bioactive compounds (using a high-throughput BMP/SMAD-driven transcriptional reporter assay) [160]. FK506 promotes BMP signaling and endothelial-specific gene regulation of genes such as APELIN. This occurs even in the absence of exogenous ligand via a dual mechanism of action: acting as an inhibitor of phosphatase CALCINEURIN and binding FK-binding protein-12 (FKBP12), a repressor of BMP signaling. FK506 promotes the release of FKBP12 from the type I receptor which leads to activation of SMAD1/5 downstream of BMP as well as MAPK signaling and ID1 gene regulation [161]. Furthermore, FK506 treatment increases ALK1 and ENDOGLIN expression in ECs [162]. Recently, a randomized, double-blind, placebo-controlled phase IIa trial was performed to investigate the efficacy of FK506 treatment in three patients with end-stage PAH. The results suggest a potential clinical benefit of low-dose FK506 (the evidence being that patients demonstrated cardiac function stabilization and required less intensive hospital care for RV failure despite the severity of the illness). It was also found that changes in serologic biomarkers indicated that BMPRII had been successfully targeted [163]. However, these results are based on a limited group of patients and the efficacy of this therapy must be validated in appropriate, well-designed clinical trials. FK506 (also known as Tacrolimus) is an immunosuppressive drug with a known pharmacokinetic and toxicity profile. It is widely used in solid organ transplantations to lower the risk of organ rejection [164]. High doses of FK506 caused systemic hypertension and transplant vasculopathy in animal models [165]. Also, organ transplant patients treated with FK506, have an increased risk of renal injury, which might occur due to the inhibition of calcineurin expression in the kidney [166–168]. In contrast, low doses of FK506 did not induce systemic hypertension in animal models, even after 3 weeks of treatment. FK506 has shown significant clinical benefits, nonetheless long-term use of this agent for treating PAH still needs to be rigorously monitored for toxicity effects.

## Conclusions and perspectives

Exogenous BMPRII delivery to ECs has been shown to be an effective means to restore BMPRII expression and function [77, 78]. An interesting approach, which is yielding promising results in mice, is to deliver BMPRII specifically to ECs using BMPRII adenoviral vectors carrying a bi-specific conjugate antibody that targets the virus to ACE, a membrane-bound protease highly expressed on pulmonary endothelial cells [77, 78]. One of the drawbacks of this strategy is the use of two components namely, adenovirus and antibody. Additional restrictions related to the use of viral transduction such as safety, specificity, and delivery of sufficient protein to revert the phenotype must also be taken into consideration. The utilization of CRISPR/Cas9 may overcome some of these limitations, for instance by minimizing the risk that the foreign gene will be integrated in the wrong place in the genome. Furthermore, it will place the gene under the control of its natural promoter. However, the delivery of CRISPR/Cas9 into the patient is still challenging and the Cas9 enzyme could cleave at unwanted locations. Similarly, the use of miRNAs targeting BMPRII has to be evaluated for off-target effects and an effective delivery system has to be found in order to consider this approach as a promising treatment. A solution for both plasmid DNA and miRNA delivery might be the use of liposomes [169] or iTOP (induced transduction by osmocytosis and propanebetaine), which is an active uptake mechanism in which NaCl-mediated hyperosmolarity together with propanebetaine triggers the uptake of macromolecules [170]. Another therapeutic strategy is the use of FDA-approved drugs that have been found to be beneficial in PAH mice models or similar diseases. Ataluren, for example, allows the cellular machinery to read-through premature stop codons [115]. Although most of the *BMPR2* mutations (~70%) are non-sense mutations, not all patients will benefit from this approach. Nevertheless, further in vivo studies are worth pursuing in the context of PAH. Likewise, clinical trials using chloroquine have to be performed to test its effectiveness in PAH patients. The use of chloroquine has to be carefully evaluated because blocking lysosomal degradation might trigger non-specific off-target effects when used as a long-term treatment. An alternative drug showing significant clinical benefits for PAH is FK506/Tacrolimus. However, it still needs to be monitored for side effects since it is an immunosuppressive drug (currently utilized after allogeneic organ transplant). Moreover, the effectiveness of FK506 at low doses has to be rigorously tested.

It is important to highlight that although several drugs showed beneficial outcomes in animal models, most of the drugs have failed in the clinic. In light of this, we should focus on a more personalized approach which takes into account the co-existence of modifier genes, infections, toxic exposure, inflammation, or alterations in estrogen metabolism. Combining treatments which target not only BMPRII signaling but also inflammation and hypoxia should improve outcomes. Lastly, the use of human ex vivo models such as lung or vessel on a chip [171] could be beneficial for drug discovery and efficacy testing in the context of PAH. We anticipate that such models may improve the relevance of pre-clinical results by using patient derived cells, especially since animal models of PAH are frequently difficult to translate into clinical practice.

Taken together, previously discussed data suggest that modulation of BMPRII signaling in PAH is a promising alternative that could prevent and reverse pulmonary vascular remodeling. However, different therapeutic approaches aimed at to increasing the levels of BMPRII signaling are needed, and these approaches will depend on the particular genetic background of each patient. In addition, for more efficient treatments, targeting other genetic and environmental factors that contribute to the disease must be taken into consideration. In this regard, modulators of the inflammatory response and estrogen metabolism could be used to help restore BMPRII signaling.

## Abbreviations

ACE: Angiotensin-converting enzyme
ACVRII: Activin type II receptor type
ACVRL1: Gene encoding activin receptor-like kinase
ALK: Activin receptor-like kinase
BMP: Bone morphogenetic protein
BMPRI: Bone morphogenetic protein type I receptor
BMPRII: Bone morphogenetic protein type II receptor
BMPR2: Gene encoding bone morphogenetic protein receptor II
Cav1: Caveolin-1
cGMP: Cyclic guanosine monophosphate
DN: Dominant negative
EC: Endothelial cell
EndMT: Endothelial-to-mesenchymal transition
FKBP12: FK-binding protein-12
GDF: Growth and differentiation factor
GDF-2: Gene encoding BMP9
HMGA1: High-mobility group protein
iTOP: Induced transduction by osmocytosis and propanebetaine
MCT: Monocrotaline
mPAP: Mean pulmonary arterial pressure
miRNA: MicroRNA
NMD: Non-sense mediated decay
PAEC: Pulmonary arterial endothelial cells
PAH: Pulmonary arterial hypertension
PASMC: Pulmonary arterial smooth muscle cells
PGI_2_: Prostacyclin
PKG: Protein kinase G
PTC: Premature termination codon
RV: Right ventricle
SMC: Smooth muscle cell
TGF-β: Transforming growth factor-beta
TNFα: Tumor necrosis factor-alfa

## References

[1] Rosenkranz S (2015). Pulmonary hypertension 2015: current definitions, terminology, and novel treatment options. Clin Res Cardiol.

[2] Guignabert C, Dorfmuller P (2013). Pathology and pathobiology of pulmonary hypertension. Semin Respir Crit Care Med.

[3] Farber HW, Loscalzo J (2004). Pulmonary arterial hypertension. N Engl J Med.

[4] Thompson K, Rabinovitch M (1996). Exogenous leukocyte and endogenous elastases can mediate mitogenic activity in pulmonary artery smooth muscle cells by release of extracellular-matrix bound basic fibroblast growth factor. J Cell Physiol.

[5] Lane KB, Machado RD, Pauciulo MW, Thomson JR, Phillips JA, Loyd JE, Nichols WC, Trembath RC, Consortium IP (2000). Heterozygous germline mutations in BMPR2, encoding a TGF-beta receptor, cause familial primary pulmonary hypertension. Nat Genet.

[6] Deng Z, Morse JH, Slager SL, Cuervo N, Moore KJ, Venetos G, Kalachikov S, Cayanis E, Fischer SG, Barst RJ, Hodge SE, Knowles JA (2000). Familial primary pulmonary hypertension (gene PPH1) is caused by mutations in the bone morphogenetic protein receptor-II gene. Am J Hum Genet.

[7] Machado RD, Aldred MA, James V, Harrison RE, Patel B, Schwalbe EC, Gruenig E, Janssen B, Koehler R, Seeger W, Eickelberg O, Olschewski H, Elliott CG, Glissmeyer E, Carlquist J, Kim M, Torbicki A, Fijalkowska A, Szewczyk G, Parma J, Abramowicz MJ, Galie N, Morisaki H, Kyotani S, Nakanishi N, Morisaki T, Humbert M, Simonneau G, Sitbon O, Soubrier F, Coulet F, Morrell NW, Trembath RC (2006). Mutations of the TGF-beta type II receptor BMPR2 in pulmonary arterial hypertension. Hum Mutat.

[8] Thomson JR, Machado RD, Pauciulo MW, Morgan NV, Humbert M, Elliott GC, Ward K, Yacoub M, Mikhail G, Rogers P, Newman J, Wheeler L, Higenbottam T, Gibbs JS, Egan J, Crozier A, Peacock A, Allcock R, Corris P, Loyd JE, Trembath RC, Nichols WC (2000). Sporadic primary pulmonary hypertension is associated with germline mutations of the gene encoding BMPR-II, a receptor member of the TGF-beta family. J Med Genet.

[9] Machado RD, Southgate L, Eichstaedt CA, Aldred MA, Austin ED, Best DH, Chung WK, Benjamin N, Elliott CG, Eyries M, Fischer C, Gräf S, Hinderhofer K, Humbert M, Keiles SB, Loyd JE, Morrell NW, Newman JH, Soubrier F, Trembath RC, Viales RR, Grünig E (2015). Pulmonary arterial hypertension: a current perspective on established and emerging molecular genetic defects. Hum Mutat.

[10] Cogan J, Austin E, Hedges L, Womack B, West J, Loyd J, Hamid R (2012). Role of BMPR2 alternative splicing in heritable pulmonary arterial hypertension penetrance. Circulation.

[11] Guignabert C, Bailly S, Humbert M (2017). Restoring BMPRII functions in pulmonary arterial hypertension: opportunities, challenges and limitations. Expert Opin Ther Targets.

[12] Song Y, Coleman L, Shi J, Beppu H, Sato K, Walsh K, Loscalzo J, Zhang Y-Y (2008). Inflammation, endothelial injury, and persistent pulmonary hypertension in heterozygous BMPR2-mutant mice. Am J Physiol Heart Circ Physiol.

[13] Austin ED, Cogan JD, West JD, Hedges LK, Hamid R, Dawson EP, Wheeler LA, Parl FF, Loyd JE, Phillips JA (2009). Alterations in oestrogen metabolism: implications for higher penetrance of familial pulmonary arterial hypertension in females. Eur Respir J.

[14] Mair KM, Yang XD, Long L, White K, Wallace E, Ewart M-A, Docherty CK, Morrell NW, MacLean MR (2015). Sex affects bone morphogenetic protein type II receptor signaling in pulmonary artery smooth muscle cells. Am J Respir Crit Care Med.

[15] Brock M, Trenkmann M, Gay RE, Michel BA, Gay S, Fischler M, Ulrich S, Speich R, Huber LC (2009). Interleukin-6 modulates the expression of the bone morphogenic protein receptor type II through a novel STAT3-microRNA cluster 17/92 pathway. Circ Res.

[16] Kim CW, Song H, Kumar S, Nam D, Kwon HS, Chang KH, Son DJ, Kang DW, Brodie SA, Weiss D, Vega JD, Alberts-Grill N, Griendling K, Taylor WR, Jo H (2013). Anti-inflammatory and antiatherogenic role of BMP receptor II in endothelial cells. Arterioscler Thromb Vasc Biol.

[17] Soon E, Crosby A, Southwood M, Yang P, Tajsic T, Toshner M, Appleby S, Shanahan CM, Bloch KD, Pepke-Zaba J, Upton P, Morrell NW (2015). Bone morphogenetic protein receptor type II deficiency and increased inflammatory cytokine production. A gateway to pulmonary arterial hypertension. Am J Respir Crit Care Med.

[18] Burton VJ, Ciuclan LI, Holmes AM, Rodman DM, Walker C, Budd DC (2011). Bone morphogenetic protein receptor II regulates pulmonary artery endothelial cell barrier function. Blood.

[19] Frumkin LR (2012). The pharmacological treatment of pulmonary arterial hypertension. Pharmacol Rev.

[20] Perrin S, Chaumais M-C, O’Connell C, Amar D, Savale L, Jaïs X, Montani D, Humbert M, Simonneau G, Sitbon O (2015). New pharmacotherapy options for pulmonary arterial hypertension. Expert Opin Pharmacother.

[21] Morrell NW, Archer SL, Defelice A, Evans S, Fiszman M, Martin T, Saulnier M, Rabinovitch M, Schermuly R, Stewart D, Truebel H, Walker G, Stenmark KR (2013). Anticipated classes of new medications and molecular targets for pulmonary arterial hypertension. Pulm Circ.

[22] Stenmark KR, Rabinovitch M (2010). Emerging therapies for the treatment of pulmonary hypertension. Pediatr Crit Care Med.

[23] Balliga RS, MacAllister RJ, Hobbs AJ (2011). New perspectives for the treatment of pulmonary hypertension. Br J Pharmacol.

[24] Liu Y, Ren W, Warburton R, Toksoz D, Fanburg BL (2009). Serotonin induces Rho/ROCK-dependent activation of Smads 1/5/8 in pulmonary artery smooth muscle cells. FASEB J.

[25] Akhurst RJ, Padgett RW (2015). Matters of context guide future research in TGFβ superfamily signaling. Sci Signal.

[26] Sánchez-Duffhues G, Hiepen C, Knaus P, Ten Dijke P (2015). Bone morphogenetic protein signaling in bone homeostasis. Bone.

[27] Morrell NW, Bloch DB, Ten Dijke P, Goumans M-JTH, Hata A, Smith J, Yu PB, Bloch KD (2016). Targeting BMP signalling in cardiovascular disease and anaemia. Nat Rev Cardiol.

[28] Kawabata M, Imamura T, Miyazono K (1998). Signal transduction by bone morphogenetic proteins. Cytokine Growth Factor Rev.

[29] Miyazono K, Kamiya Y, Morikawa M (2010). Bone morphogenetic protein receptors and signal transduction. J Biochem.

[30] Heldin CH, Miyazono K, Ten Dijke P (1997). TGF-beta signalling from cell membrane to nucleus through SMAD proteins. Nature.

[31] Derynck R, Zhang YE (2003). Smad-dependent and Smad-independent pathways in TGF-beta family signalling. Nature.

[32] David L, Feige J-J, Bailly S (2009). Emerging role of bone morphogenetic proteins in angiogenesis. Cytokine Growth Factor Rev.

[33] Feng X-H, Derynck R (2005). Specificity and versatility in tgf-beta signaling through Smads. Annu Rev Cell Dev Biol.

[34] Shi Y, Massagué J (2003). Mechanisms of TGF-beta signaling from cell membrane to the nucleus. Cell.

[35] Zhang YE (2009). Non-Smad pathways in TGF-beta signaling. Cell Res.

[36] Moustakas A, Heldin C-H (2005). Non-Smad TGF-beta signals. J Cell Sci.

[37] Mu Y, Gudey SK, Landström M (2012). Non-Smad signaling pathways. Cell Tissue Res.

[38] Massagué J, Wotton D (2000). Transcriptional control by the TGF-beta/Smad signaling system. EMBO J.

[39] Mulder KM (2000). Role of ras and mapks in TGFbeta signaling. Cytokine Growth Factor Rev.

[40] Canalis E, Economides AN, Gazzerro E (2003). Bone morphogenetic proteins, their antagonists, and the skeleton. Endocr Rev.

[41] Sánchez-Duffhues G, Hiepen C, Knaus P, Ten Dijke P (2016). Emerging regulators of BMP bioavailability. Bone.

[42] Cai J, Pardali E, Sánchez-Duffhues G, Ten Dijke P (2012). BMP signaling in vascular diseases. FEBS Lett.

[43] Gao X, Cao Y, Staloch DA, Gonzales MA, Aronson JF, Chao C, Hellmich MR, Ko TC (2014). Bone morphogenetic protein signaling protects against cerulein-induced pancreatic fibrosis. PLoS ONE.

[44] Chen NY, S DC, Luo F, Weng T, Le TT, A MH, Philip K, Molina JG, Garcia-Morales LJ, Cao Y, Ko TC, Amione-Guerra J, Al-Jabbari O, Bunge RR, Youker K, Bruckner BA, Hamid R, Davies J, Sinha N, Karmouty-Quintana H (2016). Macrophage bone morphogenic protein receptor 2 depletion in idiopathic pulmonary fibrosis and Group III pulmonary hypertension. Am J Physiol Lung Cell Mol Physiol.

[45] De Vinuesa AG, Abdelilah-Seyfried S, Knaus P, Zwijsen A, Bailly S (2016). BMP signaling in vascular biology and dysfunction. Cytokine Growth Factor Rev.

[46] Stenmark KR, Davie N, Frid M, Gerasimovskaya E, Das M (2006). Role of the adventitia in pulmonary vascular remodeling. Physiology (Bethesda).

[47] Humbert M, Morrell NW, Archer SL, Stenmark KR, MacLean MR, Lang IM, Christman BW, Weir EK, Eickelberg O, Voelkel NF, Rabinovitch M (2004). Cellular and molecular pathobiology of pulmonary arterial hypertension. J Am Coll Cardiol.

[48] Lilly B (2014). We have contact: endothelial cell-smooth muscle cell interactions. Physiology (Bethesda).

[49] Christman BW, McPherson CD, Newman JH, King GA, Bernard GR, Groves BM, Loyd JE (1992). An imbalance between the excretion of thromboxane and prostacyclin metabolites in pulmonary hypertension. N Engl J Med.

[50] David L, Mallet C, Mazerbourg S, Feige J-J, Bailly S (2007). Identification of BMP9 and BMP10 as functional activators of the orphan activin receptor-like kinase 1 (ALK1) in endothelial cells. Blood.

[51] Scharpfenecker M, van Dinther M, Liu Z, van Bezooijen RL, Zhao Q, Pukac L, Löwik CWGM, Ten Dijke P (2007). BMP-9 signals via ALK1 and inhibits bFGF-induced endothelial cell proliferation and VEGF-stimulated angiogenesis. J Cell Sci.

[52] Upton PD, Long L, Trembath RC, Morrell NW (2008). Functional characterization of bone morphogenetic protein binding sites and Smad1/5 activation in human vascular cells. Mol Pharmacol.

[53] Shintani M, Yagi H, Nakayama T, Saji T, Matsuoka R (2009). A new nonsense mutation of SMAD8 associated with pulmonary arterial hypertension. J Med Genet.

[54] Nasim MT, Ogo T, Ahmed M, Randall R, Chowdhury HM, Snape KM, Bradshaw TY, Southgate L, Lee GJ, Jackson I, Lord GM, Gibbs JS, Wilkins MR, Ohta-Ogo K, Nakamura K, Girerd B, Coulet F, Soubrier F, Humbert M, Morrell NW, Trembath RC, Machado RD (2011). Molecular genetic characterization of SMAD signaling molecules in pulmonary arterial hypertension. Hum Mutat.

[55] Wang G, Fan R, Ji R, Zou W, Penny DJ, Varghese NP, Fan Y (2016). Novel homozygous BMP9 nonsense mutation causes pulmonary arterial hypertension: a case report. BMC Pulm Med.

[56] Shintani M, Yagi H, Nakayama T, Saji T, Matsuoka R (2009). A new nonsense mutation of SMAD8 associated with pulmonary arterial hypertension. J Med Genet.

[57] Harrison RE, Berger R, Haworth SG, Tulloh R, Mache CJ, Morrell NW, Aldred MA, Trembath RC (2005). Transforming growth factor-β receptor mutations and pulmonary arterial hypertension in childhood. Circulation.

[58] Girerd B, Montani D, Coulet F, Sztrymf B, Yaici A, Jais X, Tregouet D, Reis A, Drouin-Garraud V, Fraisse A, Sitbon O, O’Callaghan DS, Simonneau G, Soubrier F, Humbert M (2010). Clinical outcomes of pulmonary arterial hypertension in patients carrying an ACVRL1 (ALK1) mutation. Am J Respir Crit Care Med.

[59] Pousada G, Baloira A, Fontan D, Nunez M, Valverde D (2016). Mutational and clinical analysis of the ENG gene in patients with pulmonary arterial hypertension. BMC Genet.

[60] Austin ED, Ma L, LeDuc C, Berman Rosenzweig E, Borczuk A, Phillips JA, Palomero T, Sumazin P, Kim HR, Talati MH, West J, Loyd JE, Chung WK (2012). Whole exome sequencing to identify a novel gene (caveolin-1) associated with human pulmonary arterial hypertension. Circ Cardiovasc Genet.

[61] Ma L, Roman-Campos D, Austin ED, Eyries M, Sampson KS, Soubrier F, Germain M, Trégouët D-A, Borczuk A, Rosenzweig EB, Girerd B, Montani D, Humbert M, Loyd JE, Kass RS, Chung WK (2013). A novel channelopathy in pulmonary arterial hypertension. N Engl J Med.

[62] Ranchoux B, Antigny F, Rucker-Martin C, Hautefort A, Pechoux C, Bogaard HJ, Dorfmuller P, Remy S, Lecerf F, Plante S, Chat S, Fadel E, Houssaini A, Anegon I, Adnot S, Simonneau G, Humbert M, Cohen-Kaminsky S, Perros F (2015). Endothelial-to-mesenchymal transition in pulmonary hypertension. Circulation.

[63] Hopper RK, Moonen JR, Diebold I, Cao A, Rhodes CJ, Tojais NF, Hennigs JK, Gu M, Wang L, Rabinovitch M (2016). In pulmonary arterial hypertension, reduced BMPR2 promotes endothelial-to-mesenchymal transition via HMGA1 and its target slug. Circulation.

[64] Maron BA, Loscalzo J (2013). Pulmonary hypertension: pathophysiology and signaling pathways. Handb Exp Pharmacol.

[65] Voelkel NF, Tuder RM (2000). Hypoxia-induced pulmonary vascular remodeling: a model for what human disease?. J Clin Invest.

[66] Campian ME, Hardziyenka M, Michel MC, Tan HL (2006). How valid are animal models to evaluate treatments for pulmonary hypertension?. Naunyn Schmiedebergs Arch Pharmacol.

[67] Guihaire J, Bogaard HJ, Flecher E, Noly PE, Mercier O, Haddad F, Fadel E (2013). Experimental models of right heart failure: a window for translational research in pulmonary hypertension. Semin Respir Crit Care Med.

[68] West J, Fagan K, Steudel W, Fouty B, Lane K, Harral J, Hoedt-Miller M, Tada Y, Ozimek J, Tuder R, Rodman DM (2004). Pulmonary hypertension in transgenic mice expressing a dominant-negative BMPRII gene in smooth muscle. Circ Res.

[69] Hong K-H, Lee YJ, Lee E, Park SO, Han C, Beppu H, Li E, Raizada MK, Bloch KD, Oh SP (2008). Genetic ablation of the BMPR2 gene in pulmonary endothelium is sufficient to predispose to pulmonary arterial hypertension. Circulation.

[70] Long L, Ormiston ML, Yang X, Southwood M, Graf S, Machado RD, Mueller M, Kinzel B, Yung LM, Wilkinson JM, Moore SD, Drake KM, Aldred MA, Yu PB, Upton PD, Morrell NW (2015). Selective enhancement of endothelial BMPR-II with BMP9 reverses pulmonary arterial hypertension. Nat Med.

[71] Maarman G, Lecour S, Butrous G, Thienemann F, Sliwa K (2013). A comprehensive review: the evolution of animal models in pulmonary hypertension research; are we there yet?. Pulm Circ.

[72] West J, Harral J, Lane K, Deng Y, Ickes B, Crona D, Albu S, Stewart D, Fagan K (2008). Mice expressing BMPR2R899X transgene in smooth muscle develop pulmonary vascular lesions. Am J Physiol Lung Cell Mol Physiol.

[73] de Jesus Perez VA, Alastalo T-P, Wu JC, Axelrod JD, Cooke JP, Amieva M, Rabinovitch M (2009). Bone morphogenetic protein 2 induces pulmonary angiogenesis via Wnt-beta-catenin and Wnt-RhoA-Rac1 pathways. J Cell Biol.

[74] Hansmann G, de Jesus Perez VA, Alastalo T-P, Alvira CM, Guignabert C, Bekker JM, Schellong S, Urashima T, Wang L, Morrell NW, Rabinovitch M (2008). An antiproliferative BMP-2/PPARgamma/apoE axis in human and murine SMCs and its role in pulmonary hypertension. J Clin Invest.

[75] Sung YK, Yuan K, de Jesus Perez VA (2016). Novel approaches to pulmonary arterial hypertension drug discovery. Expert Opin Drug Discov.

[76] Madonna R, Cocco N (2016). Novel strategies in the treatment of pulmonary arterial hypertension. Curr Drug Targets.

[77] Reynolds AM, Xia W, Holmes MD, Hodge SJ, Danilov S, Curiel DT, Morrell NW, Reynolds PN (2007). Bone morphogenetic protein type 2 receptor gene therapy attenuates hypoxic pulmonary hypertension. Am J Physiol Lung Cell Mol Physiol.

[78] Reynolds AM, Holmes MD, Danilov SM, Reynolds PN (2012). Targeted gene delivery of BMPR2 attenuates pulmonary hypertension. Eur Respir J.

[79] Harper RL, Reynolds AM, Bonder CS, Reynolds PN (2016). BMPR2 gene therapy for PAH acts via Smad and non-Smad signalling. Respirology.

[80] McMurtry MS, Moudgil R, Hashimoto K, Bonnet S, Michelakis ED, Archer SL (2007). Overexpression of human bone morphogenetic protein receptor 2 does not ameliorate monocrotaline pulmonary arterial hypertension. Am J Physiol Lung Cell Mol Physiol.

[81] Doi K, Takeuchi Y (2015). Gene therapy using retrovirus vectors: vector development and biosafety at clinical trials. Uirusu.

[82] Reynolds PN (2011). Viruses in pharmaceutical research: pulmonary vascular disease. Mol Pharm.

[83] Brunetti-Pierri N, Ng T, Iannitti D, Cioffi W, Stapleton G, Law M, Breinholt J, Palmer D, Grove N, Rice K, Bauer C, Finegold M, Beaudet A, Mullins C, Ng P (2013). Transgene expression up to 7 years in nonhuman primates following hepatic transduction with helper-dependent adenoviral vectors. Hum Gene Ther.

[84] Gubrij IB, Martin SR, Pangle AK, Kurten R, Johnson LG (2014). Attenuation of monocrotaline-induced pulmonary hypertension by luminal adeno-associated virus serotype 9 gene transfer of prostacyclin synthase. Hum Gene Ther.

[85] Kim J-D, Lee A, Choi J, Park Y, Kang H, Chang W, Lee M-S, Kim J (2015). Epigenetic modulation as a therapeutic approach for pulmonary arterial hypertension. Exp Mol Med.

[86] Xu X-F, Cheng F, Du L-Z (2011) Epigenetic regulation of pulmonary arterial hypertension. Hypertens Res 34(9):981–986. doi:10.1038/hr.2011.79 10.1038/hr.2011.79 21677658

[87] Gurha P (2016). MicroRNAs in cardiovascular disease. Curr Opin Cardiol.

[88] Welten SMJ, Goossens EAC, Quax PHA, Nossent AY (2016). The multifactorial nature of microRNAs in vascular remodelling. Cardiovasc Res.

[89] Kurakula K, Goumans M-J, Ten Dijke P (2015). Regulatory RNAs controlling vascular (dys)function by affecting TGF-β family signalling. EXCLI J.

[90] Courboulin A, Ranchoux B, Cohen-Kaminsky S, Perros F, Bonnet S (2016). MicroRNA networks in pulmonary arterial hypertension: share mechanisms with cancer?. Curr Opin Oncol.

[91] Gupta S, Li L (2015). Modulation of miRNAs in pulmonary hypertension. Int J Hypertens.

[92] Weiler J, Hunziker J, Hall J (2006). Anti-miRNA oligonucleotides (AMOs): ammunition to target miRNAs implicated in human disease?. Gene Ther.

[93] Gubrij IB, Pangle AK, Pang L, Johnson LG (2016). Reversal of microrna dysregulation in an animal model of pulmonary hypertension. PLoS ONE.

[94] Courboulin A, Paulin R, Giguère NJ, Saksouk N, Perreault T, Meloche J, Paquet ER, Biardel S, Provencher S, Côté J, Simard MJ, Bonnet S (2011). Role for miR-204 in human pulmonary arterial hypertension. J Exp Med.

[95] Bienertova-Vasku J, Novak J, Vasku A (2015). MicroRNAs in pulmonary arterial hypertension: pathogenesis, diagnosis and treatment. J Am Soc Hypertens.

[96] Fujita Y, Takeshita F, Kuwano K, Ochiya T (2013). RNAi therapeutic platforms for lung diseases. Pharmaceuticals.

[97] Comer BS, Ba M, Singer CA, Gerthoffer WT (2015). Epigenetic targets for novel therapies of lung diseases. Pharmacol Ther.

[98] Zhang Y, Wang Z, Gemeinhart RA (2013). Progress in microRNA delivery. J Control Release.

[99] Bobbin ML, Rossi JJ (2016). RNA interference (RNAi)-based therapeutics: delivering on the promise?. Annu Rev Pharmacol Toxicol.

[100] González CI, Bhattacharya A, Wang W, Peltz SW (2001). Nonsense-mediated mRNA decay in *Saccharomyces cerevisiae*. Gene.

[101] Maquat LE (2004). Nonsense-mediated mRNA decay: splicing, translation and mRNP dynamics. Nat Rev Mol Cell Biol.

[102] Noensie EN, Dietz HC (2001). A strategy for disease gene identification through nonsense-mediated mRNA decay inhibition. Nat Biotechnol.

[103] Howard M, Frizzell RA, Bedwell DM (1996). Aminoglycoside antibiotics restore CFTR function by overcoming premature stop mutations. Nat Med.

[104] Barton-Davis ER, Cordier L, Shoturma DI, Leland SE, Sweeney HL (1999). Aminoglycoside antibiotics restore dystrophin function to skeletal muscles of mdx mice. J Clin Invest.

[105] Diop D, Chauvin C, Jean-Jean O (2007). Aminoglycosides and other factors promoting stop codon readthrough in human cells. C R Biol.

[106] Kuzmiak HA, Maquat LE (2006). Applying nonsense-mediated mRNA decay research to the clinic: progress and challenges. Trends Mol Med.

[107] Hermann T (2007). Aminoglycoside antibiotics: old drugs and new therapeutic approaches. Cell Mol Life Sci.

[108] Zingman LV, Park S, Olson TM, Alekseev AE, Terzic A (2007). Aminoglycoside-induced translational read-through in disease: overcoming nonsense mutations by pharmacogenetic therapy. Clin Pharmacol Ther.

[109] Kellermayer R (2006). Translational readthrough induction of pathogenic nonsense mutations. Eur J Med Genet.

[110] Kulyté A, Dryselius R, Karlsson J, Good L (2005). Gene selective suppression of nonsense termination using antisense agents. Biochim Biophys Acta.

[111] Linde L, Kerem B (2008). Introducing sense into nonsense in treatments of human genetic diseases. Trends Genet.

[112] Namy O, Hatin I, Rousset JP (2001). Impact of the six nucleotides downstream of the stop codon on translation termination. EMBO Rep.

[113] Nasim MT, Ghouri A, Patel B, James V, Rudarakanchana N, Morrell NW, Trembath RC (2008). Stoichiometric imbalance in the receptor complex contributes to dysfunctional BMPR-II mediated signalling in pulmonary arterial hypertension. Hum Mol Genet.

[114] Hamid R, Hedges LK, Austin E, Phillips JA, Loyd JE, Cogan JD (2010). Transcripts from a novel BMPR2 termination mutation escape nonsense mediated decay by downstream translation re-initiation: implications for treating pulmonary hypertension. Clin Genet.

[115] Ryan NJ (2014). Ataluren: first global approval. Drugs.

[116] Rudarakanchana N, Flanagan JA, Chen H, Upton PD, Machado R, Patel D, Trembath RC, Morrell NW (2002). Functional analysis of bone morphogenetic protein type II receptor mutations underlying primary pulmonary hypertension. Hum Mol Genet.

[117] Rubenstein RC, Egan ME, Zeitlin PL (1997). In vitro pharmacologic restoration of CFTR-mediated chloride transport with sodium 4-phenylbutyrate in cystic fibrosis epithelial cells containing delta F508-CFTR. J Clin Invest.

[118] Rubenstein RC, Zeitlin PL (1998). A pilot clinical trial of oral sodium 4-phenylbutyrate (Buphenyl) in deltaF508-homozygous cystic fibrosis patients: partial restoration of nasal epithelial CFTR function. Am J Respir Crit Care Med.

[119] Rubenstein RC, Zeitlin PL (2000). Sodium 4-phenylbutyrate downregulates Hsc70: implications for intracellular trafficking of DeltaF508-CFTR. Am J Physiol Cell Physiol.

[120] Rubenstein RC, Lyons BM (2001). Sodium 4-phenylbutyrate downregulates HSC70 expression by facilitating mRNA degradation. Am J Physiol Lung Cell Mol Physiol.

[121] Zhang X-M, Wang X-T, Yue H, Leung SW, Thibodeau PH, Thomas PJ, Guggino SE (2003). Organic solutes rescue the functional defect in delta F508 cystic fibrosis transmembrane conductance regulator. J Biol Chem.

[122] Papp E, Csermely P (2006). Chemical chaperones: mechanisms of action and potential use. Handb Exp Pharmacol.

[123] Ma L, Liu Y, El-Achkar TM, Wu X-R (2012). Molecular and cellular effects of Tamm-Horsfall protein mutations and their rescue by chemical chaperones. J Biol Chem.

[124] Hua Y, Kandadi MR, Zhu M, Ren J, Sreejayan N (2010). Tauroursodeoxycholic acid attenuates lipid accumulation in endoplasmic reticulum-stressed macrophages. J Cardiovasc Pharmacol.

[125] da-Silva WS, Ribich S, Arrojo e Drigo R, Castillo M, Patti M-E, Bianco AC (2011). The chemical chaperones tauroursodeoxycholic and 4-phenylbutyric acid accelerate thyroid hormone activation and energy expenditure. FEBS Lett.

[126] Cao SS, Zimmermann EM, Chuang B-M, Song B, Nwokoye A, Wilkinson JE, Eaton KA, Kaufman RJ (2013). The unfolded protein response and chemical chaperones reduce protein misfolding and colitis in mice. Gastroenterology.

[127] Sobolewski A, Rudarakanchana N, Upton PD, Yang J, Crilley TK, Trembath RC, Morrell NW (2008). Failure of bone morphogenetic protein receptor trafficking in pulmonary arterial hypertension: potential for rescue. Hum Mol Genet.

[128] Yang Y-M, Lane KB, Sehgal PB (2013). Subcellular mechanisms in pulmonary arterial hypertension: combinatorial modalities that inhibit anterograde trafficking and cause bone morphogenetic protein receptor type 2 mislocalization. Pulm Circ.

[129] Frump AL, Lowery JW, Hamid R, Austin ED, de Caestecker M (2013). Abnormal trafficking of endogenously expressed BMPR2 mutant allelic products in patients with heritable pulmonary arterial hypertension. PLoS ONE.

[130] Zeitlin PL, Diener-West M, Rubenstein RC, Boyle MP, Lee CKK, Brass-Ernst L (2002). Evidence of CFTR function in cystic fibrosis after systemic administration of 4-phenylbutyrate. Mol Ther.

[131] Obici L, Cortese A, Lozza A, Lucchetti J, Gobbi M, Palladini G, Perlini S, Saraiva MJ, Merlini G (2012). Doxycycline plus tauroursodeoxycholic acid for transthyretin amyloidosis: a phase II study. Amyloid.

[132] Berry GT, Steiner RD (2001). Long-term management of patients with urea cycle disorders. J Pediatr.

[133] Iannitti T, Palmieri B (2011). Clinical and experimental applications of sodium phenylbutyrate. Drugs R D.

[134] Durrington HJ, Upton PD, Hoer S, Boname J, Dunmore BJ, Yang J, Crilley TK, Butler LM, Blackbourn DJ, Nash GB, Lehner PJ, Morrell NW (2010). Identification of a lysosomal pathway regulating degradation of the bone morphogenetic protein receptor type II. J Biol Chem.

[135] Satow R, Kurisaki A, Chan T-c, Hamazaki TS, Asashima M (2006). Dullard promotes degradation and dephosphorylation of BMP receptors and is required for neural induction. Dev Cell.

[136] Luzio JP, Pryor PR, Bright NA (2007). Lysosomes: fusion and function. Nat Rev Mol Cell Biol.

[137] Wertz JW, Bauer PM (2008). Caveolin-1 regulates BMPRII localization and signaling in vascular smooth muscle cells. Biochem Biophys Res Commun.

[138] Hartung A, Bitton-Worms K, Rechtman MM, Wenzel V, Boergermann JH, Hassel S, Henis YI, Knaus P (2006). Different routes of bone morphogenic protein (BMP) receptor endocytosis influence BMP signaling. Mol Cell Biol.

[139] Di Guglielmo GM, Le Roy C, Goodfellow AF, Wrana JL (2003). Distinct endocytic pathways regulate TGF-beta receptor signalling and turnover. Nat Cell Biol.

[140] Zaidi SH, You XM, Ciura S, O’Blenes S, Husain M, Rabinovitch M (2000). Suppressed smooth muscle proliferation and inflammatory cell invasion after arterial injury in elafin-overexpressing mice. J Clin Invest.

[141] Zaidi SHE, You X-M, Ciura S, Husain M, Rabinovitch M (2002). Overexpression of the serine elastase inhibitor elafin protects transgenic mice from hypoxic pulmonary hypertension. Circulation.

[142] Cowan KN, Heilbut A, Humpl T, Lam C, Ito S, Rabinovitch M (2000). Complete reversal of fatal pulmonary hypertension in rats by a serine elastase inhibitor. Nat Med.

[143] Alam SR, Lewis SC, Zamvar V, Pessotto R, Dweck MR, Krishan A, Goodman K, Oatey K, Harkess R, Milne L, Thomas S, Mills NM, Moore C, Semple S, Wiedow O, Stirrat C, Mirsadraee S, Newby DE, Henriksen PA (2015). Perioperative elafin for ischaemia-reperfusion injury during coronary artery bypass graft surgery: a randomised-controlled trial. Heart.

[144] Hubbard VM, Valdor R, Macian F, Cuervo AM (2012). Selective autophagy in the maintenance of cellular homeostasis in aging organisms. Biogerontology.

[145] Kroemer G, Marino G, Levine B (2010). Autophagy and the integrated stress response. Mol Cell.

[146] Long L, Yang X, Southwood M, Lu J, Marciniak SJ, Dunmore BJ, Morrell NW (2013). Chloroquine prevents progression of experimental pulmonary hypertension via inhibition of autophagy and lysosomal bone morphogenetic protein type II receptor degradation. Circ Res.

[147] Daniel JK, Fabio CA, Hagai A (2012). Guidelines for the use and interpretation of assays for monitoring autophagy. Autophagy.

[148] Ben-Zvi I, Kivity S, Langevitz P, Shoenfeld Y (2012). Hydroxychloroquine: from malaria to autoimmunity. Clin Rev Allergy Immunol.

[149] Rabinovitch M, Guignabert C, Humbert M, Nicolls MR (2014). Inflammation and immunity in the pathogenesis of pulmonary arterial hypertension. Circ Res.

[150] Ormiston ML, Upton PD, Li W, Morrell NW (2015). The promise of recombinant BMP ligands and other approaches targeting BMPR-II in the treatment of pulmonary arterial hypertension. Glob Cardiol Sci Pract.

[151] Cheng H, Jiang W, Phillips FM, Haydon RC, Peng Y, Zhou L, Luu HH, An N, Breyer B, Vanichakarn P, Szatkowski JP, Park JY, He TC (2003). Osteogenic activity of the fourteen types of human bone morphogenetic proteins (BMPs). J Bone Joint Surg Am.

[152] Croom KF, Curran MP (2008). Sildenafil: a review of its use in pulmonary arterial hypertension. Drugs.

[153] Ito T, Ozawa K, Shimada K (2007). Current drug targets and future therapy of pulmonary arterial hypertension. Curr Med Chem.

[154] Zhao L, Sebkhi A, Ali O, Wojciak-Stothard B, Mamanova L, Yang Q, Wharton J, Wilkins MR (2009). Simvastatin and sildenafil combine to attenuate pulmonary hypertension. Eur Respir J.

[155] Barnett CF, Machado RF (2006). Sildenafil in the treatment of pulmonary hypertension. Vasc Health Risk Manag.

[156] Schwappacher R, Weiske J, Heining E, Ezerski V, Marom B, Henis YI, Huber O, Knaus P (2009). Novel crosstalk to BMP signalling: cGMP-dependent kinase I modulates BMP receptor and Smad activity. EMBO J.

[157] Long L, Crosby A, Yang X, Southwood M, Upton PD, Kim D-K, Morrell NW (2009). Altered bone morphogenetic protein and transforming growth factor-beta signaling in rat models of pulmonary hypertension: potential for activin receptor-like kinase-5 inhibition in prevention and progression of disease. Circulation.

[158] Yang J, Li X, Al-Lamki RS, Wu C, Weiss A, Berk J, Schermuly RT, Morrell NW (2013). Sildenafil potentiates bone morphogenetic protein signaling in pulmonary arterial smooth muscle cells and in experimental pulmonary hypertension. Arterioscler Thromb Vasc Biol.

[159] Wang R-C, Jiang F-M, Zheng Q-l, C-T Li, Peng X-Y, C-Y He, Luo J, Liang Z-A (2014). Efficacy and safety of sildenafil treatment in pulmonary arterial hypertension: a systematic review. Respir Med.

[160] Spiekerkoetter E, Tian X, Cai J, Hopper RK, Sudheendra D, Li CG, El-Bizri N, Sawada H, Haghighat R, Chan R, Haghighat L, de Jesus Perez V, Wang L, Reddy S, Zhao M, Bernstein D, Solow-Cordero DE, Beachy PA, Wandless TJ, Ten Dijke P, Rabinovitch M (2013). FK506 activates BMPR2, rescues endothelial dysfunction, and reverses pulmonary hypertension. J Clin Invest.

[161] Wu X, Wang L, Han Y, Regan N, Li P-K, Villalona MA, Hu X, Briesewitz R, Pei D (2011). Creating diverse target-binding surfaces on FKBP12: synthesis and evaluation of a rapamycin analogue library. ACS Comb Sci.

[162] Albiñana V, Sanz-Rodríguez F, Recio-Poveda L, Bernabéu C, Botella LM (2011). Immunosuppressor FK506 increases endoglin and activin receptor-like kinase 1 expression and modulates transforming growth factor-β1 signaling in endothelial cells. Mol Pharmacol.

[163] Spiekerkoetter E, Sung YK, Sudheendra D, Bill M, Aldred MA, van de Veerdonk MC, Vonk Noordegraaf A, Long-Boyle J, Dash R, Yang PC, Lawrie A, Swift AJ, Rabinovitch M, Zamanian RT (2015). Low-dose FK506 (Tacrolimus) in end-stage pulmonary arterial hypertension. Am J Respir Crit Care Med.

[164] Plosker GL, Foster RH (2000). Tacrolimus: a further update of its pharmacology and therapeutic use in the management of organ transplantation. Drugs.

[165] Takeda Y, Miyamori I, Furukawa K, Inaba S, Mabuchi H (1999). Mechanisms of FK 506-induced hypertension in the rat. Hypertension.

[166] Drake KM, Dunmore BJ, McNelly LN, Morrell NW, Aldred MA (2013). Correction of nonsense BMPR2 and SMAD9 mutations by ataluren in pulmonary arterial hypertension. Am J Respir Cell Mol Biol.

[167] Bloom RD, Reese PP (2007). Chronic kidney disease after nonrenal solid-organ transplantation. J Am Soc Nephrol.

[168] Randhawa PS, Starzl TE, Demetris AJ (1997). Tacrolimus (FK506)-associated renal pathology. Adv Anat Pathol.

[169] Endo-Takahashi Y, Negishi Y, Nakamura A, Ukai S, Ooaku K, Oda Y, Sugimoto K, Moriyasu F, Takagi N, Suzuki R, Maruyama K, Aramaki Y (2014). Systemic delivery of miR-126 by miRNA-loaded bubble liposomes for the treatment of hindlimb ischemia. Sci Rep.

[170] D’Astolfo DS, Pagliero RJ, Pras A, Karthaus WR, Clevers H, Prasad V, Lebbink RJ, Rehmann H, Geijsen N (2015). Efficient intracellular delivery of native proteins. Cell.

[171] Esch EW, Bahinski A, Huh D (2015). Organs-on-chips at the frontiers of drug discovery. Nat Rev Drug Discov.

